# High impact of miRNA-4521 on FOXM1 expression in medulloblastoma

**DOI:** 10.1038/s41419-019-1926-1

**Published:** 2019-09-20

**Authors:** Daniel Senfter, Mahzeiar Samadaei, Robert M. Mader, Johannes Gojo, Andreas Peyrl, Georg Krupitza, Marcel Kool, Martin Sill, Christine Haberler, Gerda Ricken, Thomas Czech, Irene Slavc, Sibylle Madlener

**Affiliations:** 1 Department of Pediatrics and Adolescent Medicine, Molecular Neuro-Oncology, Medical University of Vienna, Vienna, Austria; 2 Comprehensive Cancer Center of the Medical University of Vienna, Vienna, Austria; 3 Department of Medicine III, Medical University of Vienna, Vienna, Austria; 4 Department of Medicine I, Medical University of Vienna, Vienna, Austria; 5 Department of Pathology, Medical University of Vienna, Vienna, Austria; 6 Hopp Children’s Cancer Center at the NCT Heidelberg (KiTZ), Heidelberg, Germany; 7 Division of Pediatric Neuro-Oncology, German Cancer Research Center (DKFZ), German Cancer Research Consortium (DKTK), Heidelberg, Germany; 8 Institute of Neurology, Medical University of Vienna, Vienna, Austria; 9 Department of Neurosurgery, Medical University of Vienna, Vienna, Austria

**Keywords:** CNS cancer, Apoptosis, CNS cancer

## Abstract

Medulloblastoma, an embryonal tumor of the cerebellum/fourth ventricle, is one of the most frequent malignant brain tumors in children. Although genetic variants are increasingly used in treatment stratification, survival of high-risk patients, characterized by leptomeningeal dissemination, *TP53* mutation or *MYC* amplification, is still poor. FOXM1, a proliferation-specific oncogenic transcription factor, is deregulated in various solid tumors, including medulloblastoma, and triggers cellular proliferation, migration and genomic instability. In tissue samples obtained from medulloblastoma patients, the significant upregulation of FOXM1 was associated with a loss of its putative regulating microRNA, miR-4521. To understand the underlying mechanism, we investigated the effect of miR-4521 on the expression of the transcription factor FOXM1 in medulloblastoma cell lines. Transfection of this microRNA reduced proliferation and invasion of several medulloblastoma cell lines and induced programmed cell death through activation of caspase 3/7. Further, downstream targets of FOXM1 such as PLK1 and cyclin B1 were significantly reduced thus affecting the cell cycle progression in medulloblastoma cell lines. In conclusion, a restoration of miRNA-4521 may selectively suppress the pathophysiological effect of aberrant FOXM1 expression and serve as a targeted approach for medulloblastoma therapy.

## Introduction

Brain tumors are the second most frequent neoplasms in childhood following leukemia and represent the leading cause of cancer related deaths in this age group. Among these, medulloblastoma (MB), an embryonal neoplasm arising in the cerebellum or dorsal brain stem, is one of the most frequent malignant brain tumors and accounts for up to 20% of all childhood central nervous system (CNS) tumors. Although advances in risk stratification and treatment have increased long-term survival rates for MBs over the past decades, ~30% of patients still succumb to their disease despite intensive multimodal therapy^1,2^.

In 2016, the World Health Organisation updated the classification of CNS tumors and has introduced molecular characteristics in addition to traditionally used histopathological classification^3^.

The molecular classification of MBs is based on methylome and transcriptome profiling allowing the separation into distinct molecular clusters which—by consensus—have been divided into four principal groups: Wingless (WNT)—activated, Sonic Hedgehog (SHH)-activated, and Group 3 and Group 4 MBs^1,4^. Recently, several studies suggested additional subtypes within the four groups that may allow for more accurate prediction of outcome by including additional information on gene amplifications, gene expression profiles, somatic copy number aberrations, activation of signaling pathways, and chromosomal aberrations. Loss of chromosome 17p, often accompanied by an isodicentric chromosome 17 (p11.2) created by non-allelic recombination at 17p11.2, is the most commonly detected chromosomal rearrangement in MB, detectable in 30–40% of all cases^5,6^. Patients with this chromosomal aberration tend to recur early and were found to have a worse outcome across several studies^5,6^. Although loss of tumor suppressors on 17p has been suspected to cause this effect, to date, no specific cellular mechanisms are known which could explain pathogenesis of these tumors.

microRNAs (miRNAs) are 21–25 nucleotide long non-coding RNAs, which regulate gene expression post-transcriptionally by base pairing with the 3′untranslated regions (3′UTRs) or open reading frames within the target mRNA^7–9^. Through imperfect base pairing of the specific miRNA, the target degradation is mediated by a stretch of 6–11 nucleotides. As a consequence one miRNA can target hundreds of mRNAs^10^ and induce multiple diseases including cancer. This phenomenon has also been observed in MB where epigenetic or genetic changes and chromosomal deletions or amplifications are involved in miRNA deregulation^11,12^.

FOXM1 belongs to a family of transcription factors with a shared conserved protein domain, the forkhead box (FOX), which can directly bind to DNA sequence in the enhancer region of various target genes. FOXM1 is commonly expressed in all embryonic tissues and in proliferating cells of epithelial and mesenchymal origin, but its expression is silenced after final differentiation or in non-dividing cells^13^. FOXM1 is involved in several cellular processes including chromosome segregation and G2/M transition by regulating cell cycle associated genes such as *PLK1, AURKB, CDC25B, CENPA, CENPB, CENPF*, and *CCNB1*^14–17^. A deregulation of FOXM1 was found in MB and various cancer types (i.e., liver, breast, lung, respectively) where an overexpression of FOXM1 may serve as a biomarker and correlates with poor patient outcome^16–19^.

Herein, we show for the first time a link between the aggressiveness of MB cells with a miR-4521 loss and subsequent upregulation of the oncogenic transcription factor FOXM1. Moreover, we elucidate that the resulting effects on cellular signaling and downstream targets are based on downregulation of FOXM1 protein expression.

## Results

### miR-4521 expression is downregulated in MB tissue samples and in cell lines

**Fig. 1:**
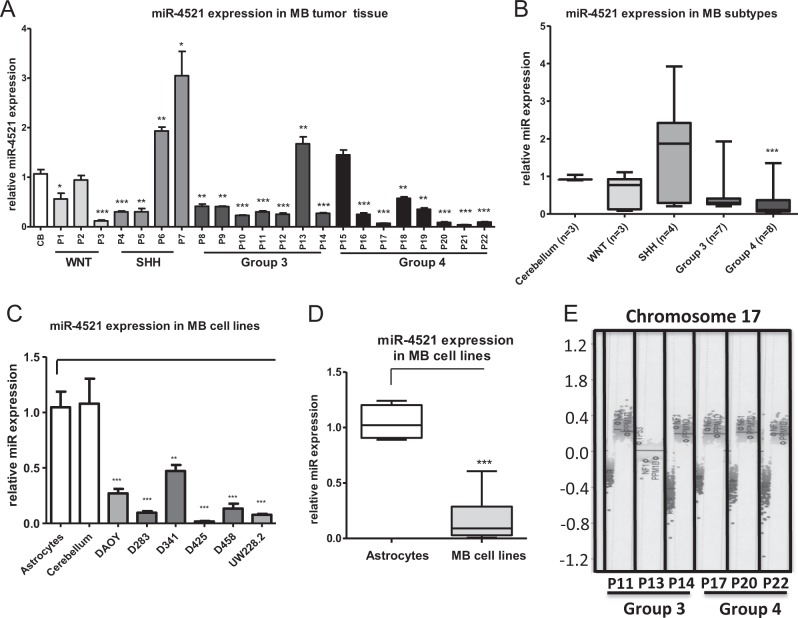
miR-4521 expression levels in MB patient tissue and MB cell lines. **a** Total RNA was isolated from MB patient tissue samples (*n* = 22). A qPCR was performed for miR-4521. A cerebellum medley (pool of 3) was used as a control. **b** The different patients were grouped according to their molecular subgroup. **c** A qPCR was performed for miR-4521 and RNU6B in different MB cell lines. Primary astrocytes were used as a control. **d** Box plot of MB cell lines compared to primary astrocytes. All qPCR data were normalized to RNU6B levels. **e** Methylation Array of MB patients—in detail, the results for chromosome 17 of representative patients. All experiments were performed in triplicates. Asterisks indicate significance (Mann–Whitney test; *P < 0.05, ***P < 0.001), error bars indicate mean ± S.D

Using in-silico analysis of different miRNA databases (targetscan and mirBase), we identified 25 miRNAs located on the p arm of chromosome 17. We focused on miRNA-4521 (miR-4521), an uncharted miRNA located on chromosome 17p13.1^20,21^. This location is also associated with the tumor suppressor gene *TP53*^22^. First, we investigated the expression of miR-4521 in our MB patient cohort (*n* = 22), including cases of all four subgroups, and compared the expression with normal cerebellum (*n* = 3) (Table 1, Fig. 1a). Seventeen out of 22 MB tumors showed a significantly lower miR-4521 expression in comparison to the control cerebellum. Grouping the samples according to the molecular classification showed that all subgroups except the SHH subgroup for which the variation was particularly wide, showed a reduction of miR-4521 expression in comparison to healthy control tissue. Only for Group 4 this difference in expression was statistically significant (Fig. 1b). To corroborate these findings in vitro, we evaluated the expression of miR-4521 in a panel of different MB cell lines belonging to SHH p53 mutated^23^ (DAOY and UW228.2) and Group 3 (D341, D425, D458) and Group 3 to 4 (D283) subgroup^5,6,23^ and detected a significant downregulation using cerebellum tissue and normal astrocytes as a cellular control (*p* < 0.001) (Fig. 1c, d). Keeping in mind that miR-4521 is located on chromosome 17p13.1 and loss of chromosome 17p is closely associated with Group 3 and 4 MBs and SHH activated p53 mutated tumors, we checked the copy number status of chromosome 17p using data from the DNA methylation array^20^. As expected, the majority of the patients belonging to the Groups 3 and 4 displayed a loss of 17p (Table 1 and Fig. 1e—representative patients). Subsequently, we searched in different in-silico databases (TarBase V8.0 and TargetScan) for predicted targets of miR-4521. The targets with the highest prediction score were used and their RNA expression analyzed using the R2: Genomics Analysis and Visualization Platform in MB dataset (dataset Cavalli hugene11t). In our MB cohort, we could show a downregulation of miR-4521. Accordingly, we were looking for an inverse correlating mRNA, which is upregulated in MB. FOXM1 was among the top 5 predicted targets with the highest upregulation and recently described to be upregulated in MB^24^.

**Table 1:** Study cohort: patient characteristics age of diagnosis (AoD), year of diagnosis (YoD), molecular subgroup (MSG), somatic copy number aberration (chr17), tumor status and follow up time (death of disease DOD, death of other cause DOC, complete remission CR, and progressive disease PD)

Case	Age of diagnosis (years)	Gender	Year of diagnosis	Molecular subtype	17p loss	17q gain	Tumor recurrence (yes/no)	Time to recurrence (Month)	Status/follow up in month
1	11	F	2009	WNT	No	No	Yes	70	DOD,71
2	9	M	2005	WNT	No	No	Yes	59	DOD,85
3	5.5	M	2012	WNT	No	No	No	n/a	CR,71+
4	1.4	M	2008	SHH	No	No	No	n/a	CR,114+
5	3	F	2007	SHH	No	No	No	n/a	CR,121+
6	1	M	2000	SHH	No	No	Yes	11	DOD,12
7	9	M	2002	SHH	Yes	No	Yes	15	DOD,15
8	4	F	1998	Group 3	Yes	No	Yes	26	DOD,67
9	12	M	2006	Group 3	No	No	Yes	31	DOC,53
10	3	F	2003	Group 3	No	No	No	n/a	CR,167+
11	4.5	F	2012	Group 3	Yes	Yes	No	n/a	CR,68+
12	2.5	F	2013	Group 3	No	No	No	n/a	CR,58+
13	1.5	F	2012	Group 3	No	No	Yes	25	CR,67+
14	1	M	2014	Group 3	Yes	Yes	Yes	2	DOD,6
15	14	M	2009	Group 4	Yes	Yes	No	n/a	CR,101+
16	3.5	M	2008	Group 4	No	Yes	No	n/a	CR,114+
17	11	M	2014	Group 4	Yes	Yes	No	n/a	CR,43+
18	10	M	2004	Group 4	Yes	Yes	Yes	41	CR,157+
19	5	M	2004	Group 4	No	Yes	No	n/a	CR,165+
20	7.5	M	2013	Group 4	Yes	Yes	Yes	25	CR,58+
21	8	F	2013	Group 4	Yes	Yes	Yes	21	PD,53+
22	11.5	M	2008	Group 4	Yes	Yes	Yes	37	CR,117+

### FOXM1 is upregulated in MB tissue samples and in MB cell lines

**Fig. 2:**
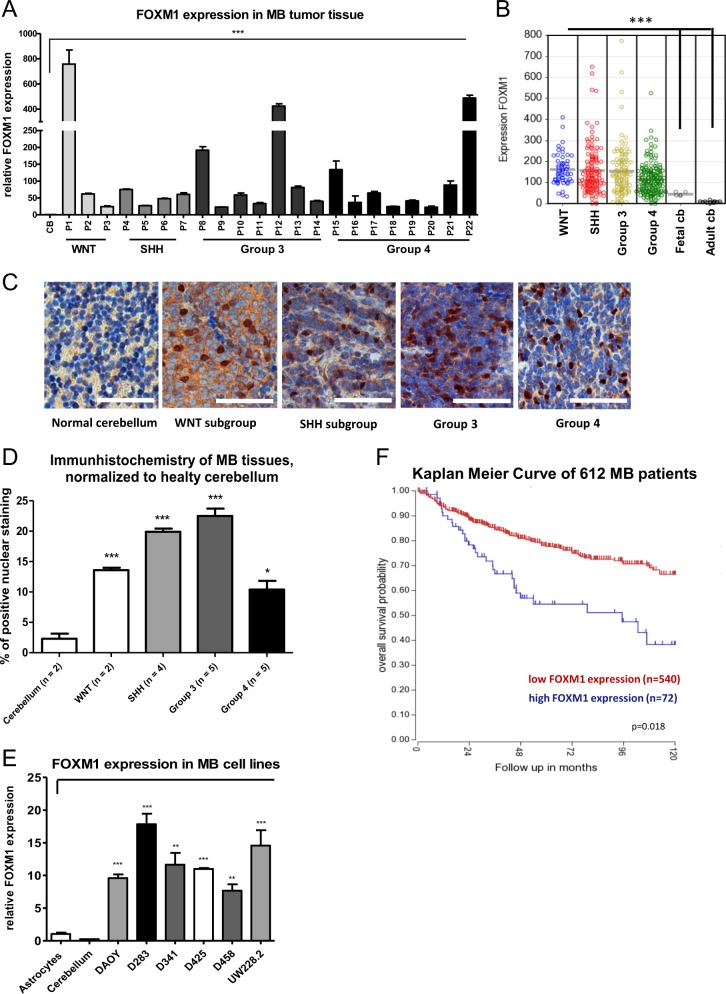
mRNA expression levels of FOXM1 in MB tissue samples and MB cell lines. **a** Total RNA was isolated from MB patient tissue samples (*n* = 22). A qPCR was performed for FOXM1 and GAPDH. A cerebellum medley (pool of 3) was used as a control. **b** Expression of FOXM1 gene in a set of 423 MB tissue samples including following molecular subgroups WNT (*n* = 53), SHH (*n* = 112), group 3 (*n* = 94) and group 4 (*n* = 164). As negative controls fetal cerebellum (*n* = 5) and adult cerebellum (*n* = 13) were used. **c** Representative images of immunohistochemical (IHC) staining of FOXM1 in MB patients (scale bar 50 µm). **d** Statistical analysis of IHC. Paraffin embedded MB tissues were stained with FOXM1, as controls a cerebellar biopsy from a 6 year old patient was used as negative control. In total, 1000 nuclei were counted and the percentage of positive stained nuclei was calculated. **e** mRNA expression levels of FOMX1 and GAPDH in different MB cell lines. Primary astrocytes were used as a control. Data were normalized to GAPDH levels. **f** Kaplan Meier curve of FOXM1 expression in 612 MB patients (R2: Genomics Analysis and Visualization Platform, dataset Cavalli hugene11t). All experiments were performed in triplicates. Asterisks indicate significance (Mann–Whitney test; *P < 0.05, ***P < 0.001), error bars indicate mean ± S.D

Subsequently, we analyzed the FOXM1 expression in our MB cohort, as well as in different MB cell lines via qPCR and detected significantly enhanced FOXM1 mRNA expression in all subgroups, with a 20 to 900-fold increase compared to healthy cerebellar tissue control (Fig. 2a). To support our data, we included gene array studies using a larger dataset of MB patient tissue samples (Affymetrix 133plus 2.0 Array, *n* = 423). This dataset confirmed our finding and showed a significant overexpression of FOXM1 compared to normal cerebellum in all MB subgroups (Fig. 2b), but no significant difference between the distinct subgroups (supplementary Fig. 1A). An immunohistochemistry staining confirmed increased levels of FOXM1 expression in MB tumors in comparison to cerebellar control (biopsy specimens of a 6-year-old and a 46-year-old patient without CNS tumor) (Fig. 2c, d). In addition, we analyzed different MB cell lines with respect to their FOXM1 mRNA expression. All cell lines showed significantly increased levels of FOXM1 (7 to 17 times increased) using primary astrocytes as control (Fig. 2e, supplementary Fig. 1B). Interestingly, in our patient cohort we observed a significantly higher expression of FOXM1 in patients who developed a tumor recurrence or responded poorly to chemotherapy when compared with patients with a good clinical outcome (supplementary Fig. 1C). To validate this in a larger series, we tested whether FOXM1 expression may correlate with overall survival of the patients using data from the Cavalli dataset^25^ (Fig. 2f). Indeed, this analysis showed that a higher FOXM1 expression in MB was significantly associated with a poorer outcome (*p* = 0.018). Thereafter, we divided the patients according to their molecular subgroups and analyzed the survival rate. In all subgroups poor outcome was associated with higher expression of FOXM1, but only in SHH and Group 4 the values were statistically significant (*p* = 0.001 and 0.05, respectively, supplementary Fig. 2A–D).

### miR-4521 decreases protein levels of FOXM1

**Fig. 3:**
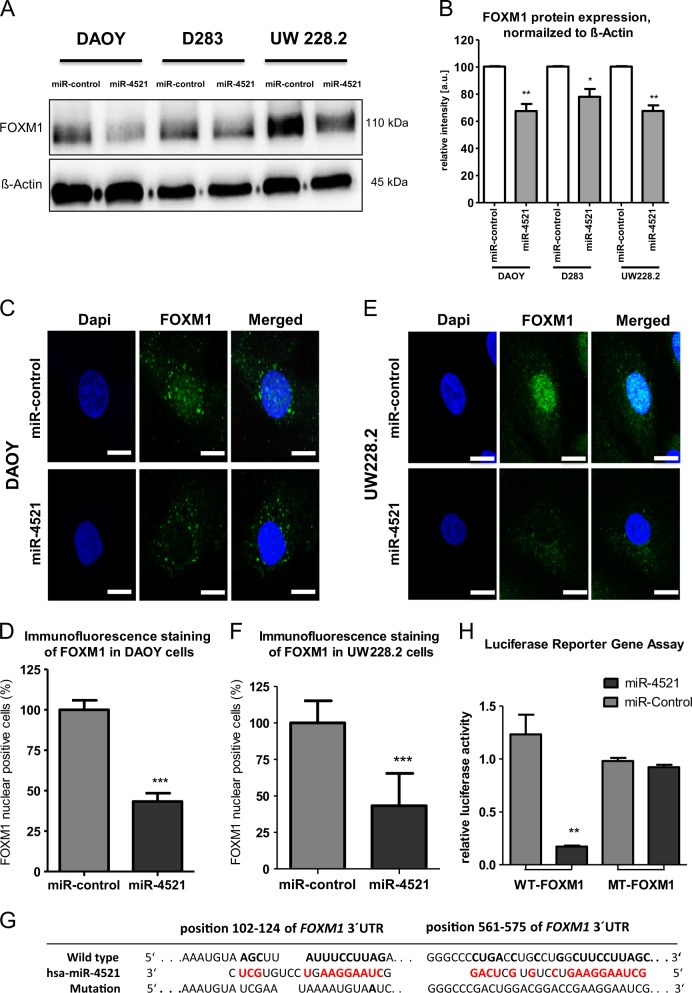
Transfection of miR-4521. MB cell lines were transfected with miR-4521 or miR-control and incubated for 72 h. **a** MB cells were lysed and western blot analysis was performed to determine the protein expression of FOXM1. ß-actin was used as loading control. All results were normalized to ß-actin and experiments were performed in triplicates. **b** Densitometry analysis of the WB. **c** miR-control or miR-4521 transfected DAOY cells were fixed 72 h after transfection and immunostained for FOXM1 (green), DNA was stained with DAPI (scale bar 20 µm). **d** Cells showing a complete translocation from nucleus to cytoplasm were quantified by counting at least 100 cells. **e** miR-control or miR-4521 transfected UW228.2 cells were fixed 72 h after transfection and immunostained for FOXM1 (green), DNA was stained with DAPI (scale bar 20 µm). **f** Cells showing a complete translocation from nucleus to cytoplasm were quantified by counting at least 100 cells. **g** Putative binding sites of miR-4521 in 3′UTR of FOXM1 mRNA. **h** Secrete-Pair Dual Luminescence Assay, WT-FOXM1 or MT-FOXM1 3′UTR was transfected into HEK cells and combined with miR-control or miR-4521-precursor. Asterisks indicate significance (students *t*-test; *P < 0.05, **P < 0.01, ***P < 0.001), error bars indicate mean ± S.D

To address the question, whether high FOXM1 and low miR-4521 expression levels were causally related, and if re-expression of miR-4521 could suppress FOXM1 in MB cell lines, we transfected DAOY, D283 and UW228.2 with a miR-4521 precursor. We could demonstrate that re-expression of miR-4521 decreased the FOXM1 protein levels by 25 to 30% (Fig. 3a, b). To prove that the FOXM1 downregulation was caused by the miRNA transfection, we verified the transfection efficiency into the MB cell lines by qPCR. Depending on the cell line, upregulation of miR-4521 ranged from 40-fold up to 230-fold increase (DAOY: 40-fold, UW228.2: 230-fold, D283: 55-fold) (supplementary Fig. 3A). In addition, a significant translocation of FOXM1 from the nucleus to the cytoplasm was observed in DAOY and UW228.2 upon transfection (Fig. 3c, e). By counting more than 100 cells of each condition, we detected a significant increase of FOXM1 nuclear positive cells in miR-control transfected cells when compared with miR-4521 transfected cells (Fig. 3d, f). In addition, we transfected DAOY and UW228.2 cells with siFOXM1 or siControl and observed the same effect on translocation in FOXM1 downregulated cells (supplementary Fig. 3D–G).To corroborate these findings, we analyzed the cytoplasmic and nuclear fraction of miR-4521 transfected cells for FOXM1 expression via western blot (supplementary Fig. 3B–C). FOXM1 was weakly detected in the cytoplasm fraction, but the nuclear fraction of the miR-4521 transfected cells showed a reduction of FOXM1 expression when compared with the control transfection.

### miR-4521 targets 3′UTR of *FOXM1* mRNA

To validate the two predicted binding sites of miR-4521 in the 3′UTR of *FOXM1* mRNA, we performed a Secrete-Pair-Dual-Luminescence Assay. The predicted binding sites of miR-4521 in the 3′UTR of FOXM1 were characterized by miRDB.org and are shown in Fig. 3g. As demonstrated in Fig. 3h, the transfected cells with WT-FOXM1-3′-UTR-pEXZ-MT05 (WT-FOXM1) in combination with miR-4521-precursor significantly downregulated the luciferase activity to 0.17 (*p* < 0.01) compared with miR-control (1.3). Mutations of the two predicted binding sites (MT-FOXM1) reversed the luciferase activity of miR-4521-precursor and were comparable to miR-control (0.98 and 1). Likewise, an overexpression of FOXM1 cDNA without 3′UTR and co-transfection with miR-4521 reveal an increase FOXM1 expression in DAOY cells (supplementary Fig. 1D–E). These results confirm the direct binding and regulation of FOXM1 via miR-4521 expression.

### Targeting FOXM1 with miR-4521 causes reduced proliferation, colony and spheroid formation of MB cells

**Fig. 4:**
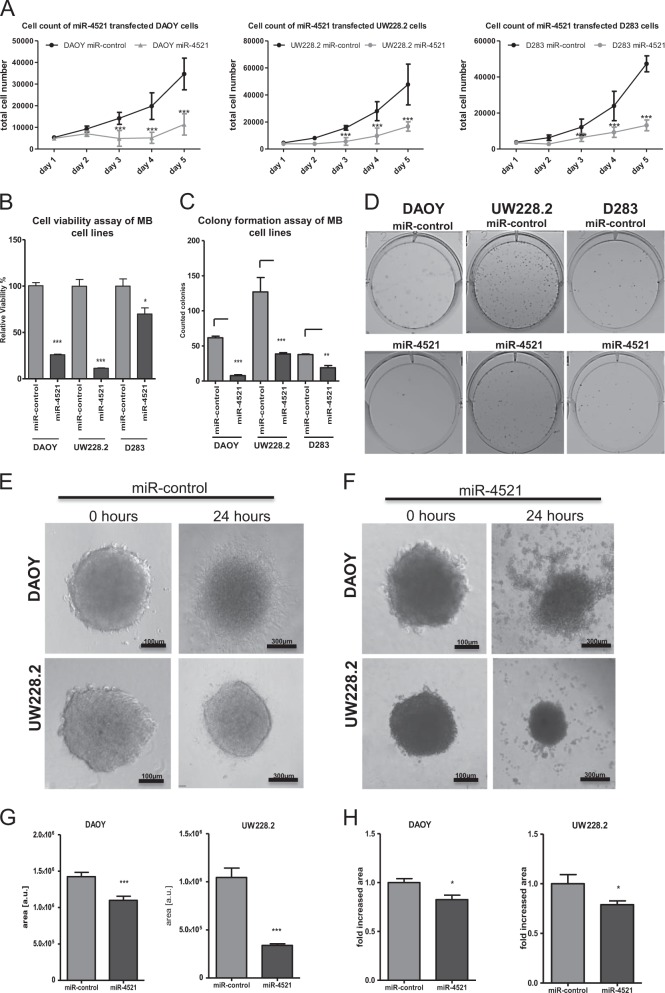
Anti-proliferative effect of miR-4521 in MB cell lines. MB cell lines were transfected with miR-4521 or miR-control. 24 h post-transfection cells were re-seeded (1 × 10^4^ cells/well). **a** Total cell number of the miR-4521 or miR-control cells is shown at different time points. **b** The relative viability of miRNA transfected cells was measured 3 days post transfection via a MTT assay. **c** A colony formation assay using miR-4521 transfected MB cells was performed (1 × 10^3^ cells/6-well). Representative pictures of the fixed and stained cells after 7 days growing. **d** The colonies of different cell lines and conditions were counted. All experiments were performed in triplicates. **e** DAOY and UW228.2 miR-control transfected cells after 0 and 24 h embedding. **f** DAOY and UW228.2 miR-4521 transfected cells after 0 and 24 h embedding. **g** The area of at least 10 spheroids was measured after embedding with Image J software. **h** The outgrowth rate of the miR-4521 transfected spheroids was calculated by comparing them to miR-control transfected spheroids after 24 h. Asterisks indicate significance (students *t*-test; *P < 0.05, ***P < 0.001), error bars indicate mean ± S.D

After showing that miR-4521 downregulates FOXM1 protein expression and affects its subcellular location, we investigated whether transfection of miR-4521 in DAOY, UW228.2 and D283 cells had an effect on cell proliferation. Upon miR-4521 transfection, cell proliferation was determined by counting cell numbers for 5 days. As illustrated in Fig. 4a, b all cell lines showed statistically significant reduced viability (*p* < 0.001). miR-4521 showed the highest effect in DAOY and UW228.2 with 75% and 90% reduced viability, respectively, whereas D283 showed a 30% reduced viability. In an independent experiment, we overexpressed FOXM1 cDNA without 3′UTR sequence through plasmid transfection combined with miR-control or miR-4521 and analyzed the growth rate after 72 h (supplementary Fig. 1E). DAOY cells reached an increased proliferation rate in comparison to GFP transfected cells. Further, we performed a colony formation assay and could demonstrate that all three cell lines (DAOY, UW228.2 and D283) showed reduced capability to form colonies (DAOY: 80%, UW228.2: 50% D283: 70% reduction) when transfected with miR-4521 (Fig. 4c, d). To demonstrate that this effect is caused by a downregulation of FOXM1, we used FOXM1 siRNA (supplementary Fig. 4C), which transfection suppressed colony formation by 40 to 60% (supplementary Fig. 4A–B). Furthermore, we investigated the effects of miR-4521 in a 3D–cell model, where miR-4521 transfected cells were grown as spheroids, embedded into a collagen gel and analyzed for their size and shape. DAOY and UW228.2 formed stable spheroids, whereas D283 spheroids were fragile and embedment into a collagen gel resulted in disruption of the spheroid. In DAOY and UW228.2 grown for 3 days post transfection, a significant decrease in size was observed (23% and 67%, respectively) (Fig. 4e–g). Likewise, miR-4521 transfected spheroids showed a reduced ability to invade into the collagen gel when compared with the miR-control transfected spheroids (Fig. 4h).

### miR-4521 influences cell cycle division and induces cell-cycle arrest through downregulation of FOXM1

The transcription factor FOXM1 is a major regulator of G1/S, G2/M cell cycle progression and is crucial for mitotic spindle integrity^26^. Upon transfection with miR-4521 or siRNA against FOXM1, we observed a significant increase of cells with spindle defect (>3 nuclei/cell) in DAOY and UW228.2 (20 to 30% and 25 to 40%), indicating an aberrant cell division in the transfected cells, whereas the control group (miR-control and siRNA-control) showed similar levels of defective nuclei (10 to 13%) (supplementary Fig. 5A–F).

Moreover, a significantly increased accumulation of cells in subG1, indicative for cell death, combined with a decrease in all other cell cycle phases after miR-4521 transfection was observed in all cell lines (supplementary Fig. 6).

### miR-4521 induces apoptosis via Caspase 3/7 pathway activation in MB cell lines

**Fig. 5:**
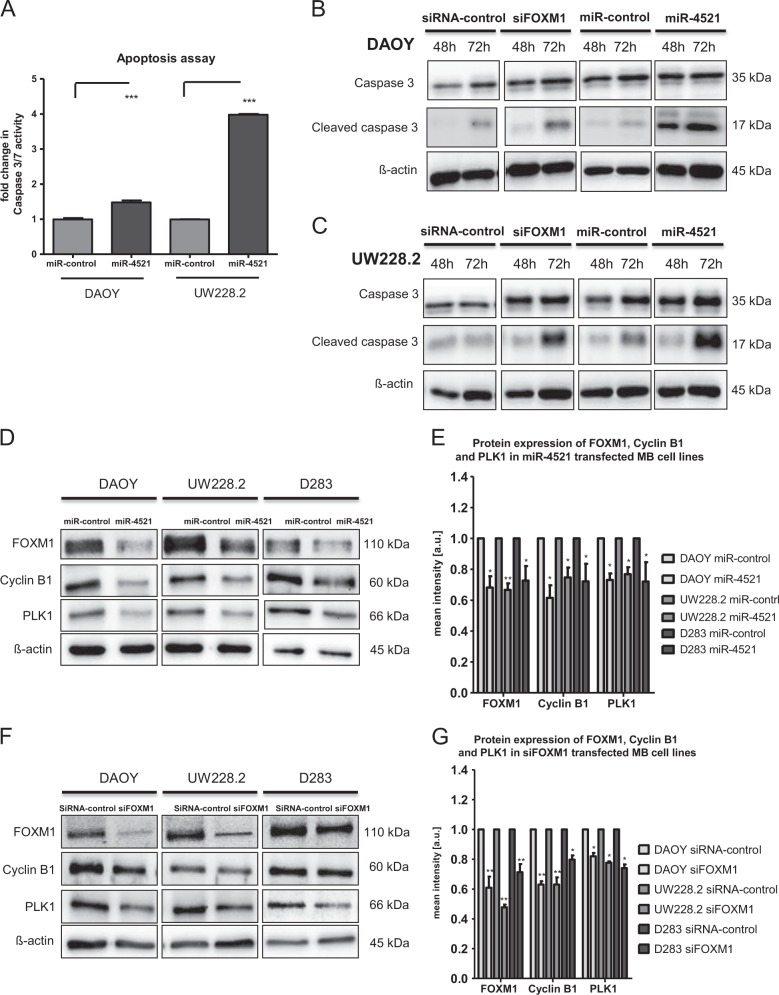
miR-4521 induces apoptosis via the caspase 3/7 axis. **a** DAOY and UW228.2 cells were transfected with miR-4521 or miR-control. After 72 h the cellular caspase 3/7 activity was measured. **b** DAOY cells were transfected with miR-control/miR-4521 or siRNA-control/siFOXM1 and protein was isolated on 2 time points, 48 h and 72 h post transfection. The levels of cleaved caspase 3 and caspase were detected three times via WB for the different conditions. **c** UW228.2 cells were transfected with miR-control/ miR-4521 or siRNA-control/ siFOXM1 and protein was isolated on 2 time points, 48 h and 72 h post transfection. The levels of cleaved caspase 3 and caspase were detected three times via WB for the different conditions. **d** DAOY, UW228.2, and D283 cells were transfected with miR-control or miR-4521 resulting in a downregulation of FOXM1 downstream targets cyclin B1 and PLK1. miR-4521 transfection downregulates FOXM1 downstream targets. **e** Densitometry analysis of three independent WB results of the miR-4521 transfected cells. ß-actin was used as a loading control and the protein expression normalized to the ß-actin levels. **f** DAOY, UW228.2, and D283 cells were transfected with siRNA-control or siFOXM1 as a control to see a downregulation of FOXM1 downstream targets cyclin B1 and PLK1. **g** Densitometry analysis of three independent WB results of the siFOXM1 transfected cells. ß-actin was used as a loading control and the protein expression normalized to the ß-actin levels. Asterisks indicate significance (students *t*-test; *P < 0.05, **P < 0.01), error bars indicate mean ± S.D

Next, we addressed the question whether these subG1 accumulated cells will be eliminated by activating an apoptosis pathway. The transfection of miR-4521 into DAOY and UW228.2 resulted in a significant activation of the apoptosis pathway via Caspase 3/7 activity (1.5 and 4-fold increased, respectively) as shown in Fig. 5a. In addition, we analyzed the cleavage of Caspase 3 through miR-4521 expression by western blotting over time. To confirm that the pro-apoptotic activity of miR-4521 was specific to FOXM1 downregulation, we performed this analysis with siFOXM1 transfected DAOY and UW228.2 cells. FOXM1 siRNA caused a significant cleavage of caspase 3 after 72 h similar to miR-4521 transfected DAOY and UW228.2 cells (Fig. 5b, c).

### Downregulation of FOXM1 via miR-4521 decreases major downstream targets of FOXM1

As mentioned before, FOXM1 is involved in several cellular processes including chromosome segregation and G2/M transition by regulating cell cycle associated genes. We focused and investigated the protein expression of two known downstream targets of FOXM1, PLK1 and cyclin B1, two major G2/M cell cycle regulators.

To examine the effect of miR-4521 transfection on FOXM1 and its targets (PLK1 and cyclin B1), miR-4521 or FOXM1 siRNA transfected MB cell lines, were analyzed by western blot. All cell lines showed a significant downregulation of FOXM1 protein via miR-4521. We observed the maximum downregulation of downstream targets of FOXM1 72 h after transfection with miR-4521 (Fig. 5d, e and supplementary Fig. 7). Analyzing the cyclin B1 and PLK1 expression by confocal microscopy in MB cell lines, we could confirm our results obtained by immunoblotting in both cell lines (supplementary Figs. 8, 9) To demonstrate that the observed effects were caused by FOXM1 downregulation, we used siRNA to specifically downregulate FOXM1 (Fig. 5f, g). We verified that miR-4521 downregulates the FOXM1 downstream targets PLK1 and cyclin B1 in the used MB cell lines. In analogy, cells transfected with siFOXM1 showed the same effect on PLK1 and cyclin B1.

## Discussion

In the presented work, we describe a tumor suppressor mechanism of miR-4521 regulating FOXM1 expression in MB. We identified and investigated for the first time the role of miR-4521, a miRNA located on chromosome arm 17p, a gene locus which is often deleted in MB. This miR-4521 was first described in 2014 to be downregulated under hypoxic conditions in a breast cancer cell line. In 2015, Zhuang linked overexpression of miR-4521 to neural differentiation of human Wharton′s Jelly mesenchymal stem cells^21,27^. Here, we show the ability of this miRNA to downregulate the aberrantly expressed transcription factor FOXM1, leading to a reduced proliferation rate, loss of colony formation, tumor invasion and induced programmed cell death through activation of the caspase 3/7 pathway in MB cells.

Chromosome 17 defects including loss of 17p and isochromosome 17q are the most common genetic aberrations observed in MB suggesting a mechanism for pathogenesis on this chromosome. We identified 25 small nucleic acids on chromosome 17p of which miR-4521, a less investigated and little-known miRNA, attracted our attention. In our patient cohort (*n* = 22), the analyzed expression levels of miR-4521 correlated with the loss of 17p determined by a DNA methylation array (Fig. 1a, e). As shown in Fig. 1e, patients with a loss of 17p showed reduced expression of miR-4521 compared to balanced chromosome 17 patient.

As a potential target of miR-4521, the databases identified FOXM1, a transcription factor which gained attention by regulating a network of genes associated with proliferation, oncogenesis and cancer progression^28^. This miRNA binds at two sites in the 3′UTR region of FOXM1 (transcript position 102–124 and 561–575) and an in silico target analysis using DIANA tool (microT_CDS) confirmed FOXM1 as a predicted and experimentally verified target of miR-4521. Here, we could confirm the regulative and specific binding of miR-4521 on FOXM1 3′UTR by a luciferase reporter assay (Fig. 3h). In our study, the transfection of miR-4521 reduced the stability of FOXM1 protein in all investigated MB cell lines (Fig. 3a, b).

In several clinical studies, a link between aberrant expression levels of the transcription factor FOXM1 and poor prognosis for many cancers including breast, liver, lung prostate, melanoma and gliomas have been described^14,16,17,28,29^. In 2011, Priller and colleagues showed that FOXM1 is significantly upregulated in MB cell lines and tumor tissue and linked the high expression level of FOXM1 to an unfavorable patient outcome^24^. In line with their findings FOXM1 was significantly upregulated in our MB patient cohort and patients with a worse clinical outcome had significantly higher levels of FOXM1 than the remaining patients (supplementary Fig. 1C). Further, a Kaplan Meier survival curve of 612 MB patients (TCGA dataset rma_sketch hugene 11t, FOXM1 expression—Cavalli et al.)^25^ supported the association of poor overall survival and FOXM1 overexpression in MB (Fig. 2f, supplementary Fig. 2A–D).

Previous studies described an increased nuclear staining of FOXM1 in human mammary tumors vs. normal tissue and correlated the FOXM1 expression to Her2 expression, resistance to chemotherapy and a poor patient outcome^30,31^. Other studies demonstrated the impact of nuclear FOXM1 localization in several cancer types^32,33^. A knock down of FOXM1 decreased nuclear translocation of FOXM1 and reduced protein concentration of beta catenin in the nucleus of colon cancer and glioma, respectively. In line with these studies, our results showed a distinct nuclear localization of FOXM1 and its translocation after transfection of miR-4521 in MB cell lines (Fig. 3)^32,34^. The translocation of FOXM1 from the nucleus to cytoplasm after miR-4521 transfection indicates the significant impact of this miRNA, decreased in MB patients due to common genetic defects on chromosome 17.

Hyper-proliferation, enhanced invasion and evading apoptosis are known hallmarks of cancer. Recent studies showed the impact of FOXM1 expression on these mechanisms^35–37^. In 2015, Wang and colleagues described the critical role of FOXM1 in the development and progression of GBM through regulation of key factors involved in proliferation or invasion, respectively^35^. Here, we showed that transfection of miR-4521 caused a significant reduction of the cellular proliferation and the loss of colony formation in all investigated MB cell lines. Further, our 3D spheres showed an excessive shrinkage and disaggregation within 24 h after restoration of miR-4521 (Fig. 4). This effect was also described after FOXM1 depletion in a previous report in breast cancer^38^.

FOXM1 is a key player in cell cycle progression and is regulating a network of proliferation associated genes that are involved in mitotic spindle assembly, G2/M transition and chromosome segregation by regulating cell cycle genes^14–17^. Here, we focused on two proteins essential for the cell cycle, i.e., PLK1 and cyclin B1. PLK1 is known as an early activator of the G2/M transition in the cell cycle and is described as a proto-oncogene and overexpressed in many different tumor types. Besides PLK1, cyclin B1 is a major cell cycle regulator protein during mitosis. Overexpression of cyclin B1 is related to uncontrolled cell cycle progression. Therefore, cyclin B1 also acts as a proto-oncogene with increased expression in a variety of tumors^39–41^. As shown in supplementary Figs. 5 and 6, miR-4521 overexpression or depletion of FOXM1 with siRNA resulted in an incomplete cell cycle progression in line with a significant decrease of PLK1 and cyclin B1 protein expression in the tested MB cell lines (Fig. 5 and supplementary Fig. 7).

Previous studies revealed the role of increased FOXM1 expression in proliferation, tumor progression and therapeutic resistance^30,42^. In line with other studies, where a downregulation of FOXM1 using siRNA or inhibitors resulted in mitotic catastrophe and apoptosis via the caspase 3/7 pathway, we observed a similar effect upon miR-4521 transfection^37,43^. We confirmed the specific killing effect of miR-4521 through cleavage of caspase 3 and activation of programmed cell death in MB cell lines (Fig. 5). The same activation was observed when FOXM1 was specifically downregulated with siRNA.

The high expression level and role of FOXM1 in MB and other cancer types make it an interesting and suitable target for clinical intervention^14,17,24^. Current research findings indicate the essential role of FOXM1 and linked the deregulation of FOXM1 to cancer progression and cancer drug resistance^19,31,42,44,45^.

Currently, development of chemical compounds to inhibit FOXM1 is underway but no approved inhibitors are available yet. Here, we tested the inhibitory and regulatory effect of the small nucleic acid 4521 on FOXM1 expression and stability.

In conclusion, we described the decreasing effect of miR-4521 on FOXM1 protein expression followed by a cellular translocation of FOXM1 protein. Further, our study demonstrated the high impact of miR-4521 expression through a reduction of the proliferation and invasion rate in MB cells. Ultimately, this miRNA activates the specific cell death through the caspase 3/7 pathway. In the future, this small miRNA that exhibits a significant growth inhibition on MB cells may provide a targeted approach to MB therapy.

## Materials and methods

### Antibodies and reagents

The primary antibodies used in our experiments were FOXM1 (Cell Signaling, D12D5, #5436, Danvers, MA, USA), FOXM1 for immunohistochemistry (Santa Cruz, C-20, sc-502, Dallas, Texas, USA), Cyclin B1 (Santa Cruz, H-433, sc-752), PLK1 (Abcam, ab17057, Cambridge, United Kingdom), Cleaved Caspase 3 (Cell Signaling, Asp175, #9664), Caspase 3 (Cell Signaling, 8G10, #9665), Histone H3 (Cell Signaling, #9715), Lamin A/C (Sigma Aldrich, SAB4200236, St. Louis, Missouri, USA) α-Tubulin (Santa Cruz, sc-8035), and β-Actin (Abcam, ab8229).

siRNA against FOXM1 (HSS103712) and a custom made negative control were purchased from Thermo Fisher Scientific. Pre-miRNA-4521 (AM17100; PM21281) and negative pre-miR control (AM17111) were purchased from Ambion (Thermo Fisher Scientific, Waltham, MA, USA). The primers which were used to detect FOXM1 and Glyceraldehyde 3-phosphate dehydrogenase (GAPDH) were ordered by IDT. For detection of miR-4521 the TaqMan system was used (Thermo Fisher Scientific, #4427975).

### Cell lines

DAOY, D283Med, D341Med short (D283 and D341) were obtained from American Type Culture Collection (ATCC, Manassas, Virginia, USA), whereas UW228.2, D425Med and D458Med short (D425 and D458) and HEK293 cells were provided by Thomas Ströbel from the Institute of Neurology Medical University of Vienna. All cells were cultured in Dulbecco’s Modified Eagle Medium (Sigma-Aldrich) containing 10% fetal bovine serum, except D341 (20% FBS) (FBS, Sigma-Aldrich), 2% L-Glutamine (Sigma-Aldrich) and 0.2% Normocin (Sigma-Aldrich) and were incubated at 37 °C in a 5% CO2 atmosphere. Prior to the experiments cells were determined to be mycoplasma negative by testing with LookOut Mycoplasma PCR detection kit (Sigma-Aldrich).

### Tumor tissue samples and molecular subgrouping of the MB tissue sample

The tumor samples were collected immediately after surgery, snap frozen and stored at −80 °C. Genomic DNA from frozen MB tissue samples was isolated using the QIAamp DNA Mini Kit according to the manufacturer’s instructions (Qiagen, Hilden, Germany). Using a 450 K DNA-methylation array the classification into the four subgroups was performed at the German Cancer Research Center (DKFZ), Heidelberg, Germany 45.

### Transfection

FOXM1 was transiently knocked down using following synthetic siRNA (5′-CCCUGCCCAACAGGAGUCUAAUCAA-3′ and 5′-UUAAUUAGACUCGUCUUGGGCAGGG-3′). FOXM1 WT overexpression experiments were performed by using a FOXM1 cDNA ORF clone fused with a C-GFPSpark-tag purchased from Sinobiological. GFP empty vector was used for transfection control and normalization. For transfection of miR4521 a pre miR-4521 (5′-GCUAAGGAAGUCCUGUGCUCAG-3′) and miR-negative control #1 were used. Cells were transfected with Lipofectamin 2000 (Invitrogen, Thermo Fisher Scientific) according to manufacturer′s manuals at the final concentration of 50–75 nM.

### Colony formation assay

Transfected cells were harvested and 1 × 10^3^ of these cells were seeded into 6-well plates and were incubated for 7 days. Afterwards, the cells were fixed with methanol followed by a crystal violet (Merck, Darmstadt, Germany) staining. The number of colonies including more than 50 cells was counted.

### Cell viability assay

1 × 10^4^ of the transfected cells were seeded in 24-well and incubated and harvested on day three and four followed by a Trypan Blue staining and counted by using a hemocytometer. In addition, a MTT assay using the CellTiter Blue Reagent (Promega, Madison, Wisconsin, USA) was performed according to the manufacturer’s protocol. Three independent experiments were performed.

### Flow cytometry analysis (FACS)

MB cells were transfected and incubated for 72 h. Afterwards, the cells were washed, pelleted and re-suspended dropwise with ice cold 85% ethanol and incubated at −20 °C for at least 30 min to fix the cells. After a washing and centrifugation step (at 4 °C for 15 min @ 1500 rpm) the cells were resuspended with a Probidium Iodide solution (10 µg/ml, Sigma Aldrich) in PBS. The cell cycle distribution was measured with a FACS Canto II and analyzed with the FACS Diva software.

### Western Blot

The different transfected MB cell lines were harvested 3 days post transfection and washed twice with cold PBS, placed on ice and lysed with a buffer containing 50 ml Tris–HCl (pH = 6.8), 6% SDS, 20% glycerol, 1.85 mM EDTA, phosphatase inhibitor cocktail and protease inhibitor cocktail. The lysate was stored at −20 °C until analysis. The separation of equal amounts of protein was performed by SDS polyacrylamide gel electrophoresis and transferred onto a PVDF-membrane (GE Healthcare, Chicago, Illinois, USA) with 100 V for 1 h in cold transfer buffer. The washed membranes were blocked for 1 h in 5% bovine serum albumin (BSA) solution in PBS with 0.1% Tween 20 (PBST), washed again with PBST and incubated with respective primary antibodies (dilution 1:500–1:1000 in TBST with 5% BSA) at 4 °C overnight. Then the membrane was washed and incubated with the secondary antibody (peroxidase-conjugated goat anti-rabbit IgG, anti-rabbit or anti-mouse IgG; dilution 1:2000–1:10000; Dako, Santa Clara, CA, USA) at room temperature for 1 h. ECL detection kit (Thermo Scientific) was used to develop chemiluminescence on membranes and were exposed in an automatic Fusion FX (Vilber Lourmat, Eberhardzell, Germany).

### Nucleus-cytoplasm fraction

The miR-4521 and control transfected cells were harvested 3 days post transfection, washed twice in PBS and prepared as described in the supplementary experimental procedure.

### Secrete-pair-dual-luminescence assay

By using the miRDB.org database we identified the two potential binding sites of miR-4521 in the 3′UTR of FOXM1 mRNA. The 3′UTR of FOXM1 was cloned by GeneCopoeia into pEXZ-MT05 clone (WT-FOXM1). For the binding control, we ordered from GeneCopoeia a mutated version of both putative binding sites of miR-4521 in the 3′UTR of FOXM1 (pEXZ-MT05-1 named here as MT FOXM1).

3 × 10^5^ HEK293 cells were seeded in a 6-well and incubated for 24 h at 37 °C in a 5% CO2 atmosphere. Thereafter, cells were transfected with 500 ng pEXZ-MT05 or pEXZ-MT05-1 in presence of 50 nM miR-4521 precursor or miR-control. After 72 h the secrete pair dual luminescence assay was performed as described in the instructor’s manual from GeneCopoeia and analyzed with the GloMax Multi + detection system from Promega.

### Caspase 3/7 activity assay

DAOY and UW228.2 cells were transfected with miR-4521 or miR-control and incubated for 72 h. Afterwards, the transfected cells were counted and equal number of cells (2 × 10^5^ per well) were transferred into a 96-well plate. The activity of Caspase 3/7 was measured by Apo-ONE® Homogeneous Caspase-3/7 Assay kit (Promega) according to the manufacturer’s instructions and analyzed by the GloMax Multi + detection system from Promega.

### Real-time PCR

RNA was isolated using Tri reagent (Sigma-Aldrich) and for detection of the miRNA the TaqMan system was chosen. Ten nanogram RNA was used for reverse transcription using the TaqMan MicroRNA RT Kit (Applied Biosystems, Life Technologies). For the detection of FOXM1 and GAPDH the SYBR-green system was used. The detailed description is written in the supplementary experimental procedures.

### Spheroid invasion assay

For creating spheroids the hanging drop method was performed as described in the supplementary experimental procedure.

### Immunofluorescence (IF)

DAOY and UW228.2 cells were grown on cover slips and fixed with methanol after transfection with miR-4521 or FOXM1 siRNA. After fixation of the cells, methanol was removed and cells were blocked with 5% BSA/PBS solution for 1 h at room temperature. The dilution of the first antibody was chosen according to the datasheet of the antibody and incubated in a wet chamber over night at 4 °C. Afterwards, the coverslips containing the fixed cells were washed three times with PBS and incubated with the secondary Alexa Fluor 488 or Alexa Fluor 594 labeled antibody (1:1000 dilution) for 1 h at room temperature. The stained slides were mounted on Vectashield DAPI mounting medium. The immunofluorescence was performed by using a Zeiss confocal microscope LSM780 (Zeiss, Oberkochen, Germany) and analyzed with Image J software.

### Immunohistochemistry

Formalin-fixed, paraffin-embedded tissue sections (3 µm) were used for immunohistochemistry as described in the supplementary experimental procedure.

### Statistical analysis

All experiments were carried out in triplicates. A statistical analysis of the experiments was performed using the GraphPad prism 5 software. The values are represented as ± the standard deviation (S.D). Before calculating the *p*-values, a column statistics was performed to test if the data passes the normality test. For data which passed the normality test the Student’s *t*-test was used and for all others we applied the Mann–Whitney test. The *p*-values indicate statistical significance (*P < 0.05, **P < 0.01, ***P < 0.001).

## Supplementary information

Supplementary Figures

Supplementary file
