# Supplementary material for: High impact of miRNA-4521 on FOXM1 expression in medulloblastoma
Source: Cell Death Dis. 2019 Sep 20;10(10):696. doi: 10.1038/s41419-019-1926-1 (PMC6754377; doi:10.1038/s41419-019-1926-1)
Supplement: Supplementary file 1 — Supplementary Figures [file 41419_2019_1926_MOESM1_ESM.pdf]

SUPPLEMENTAL FIGURE AND FIGURE LEGENDS

Supplementary Figure 1

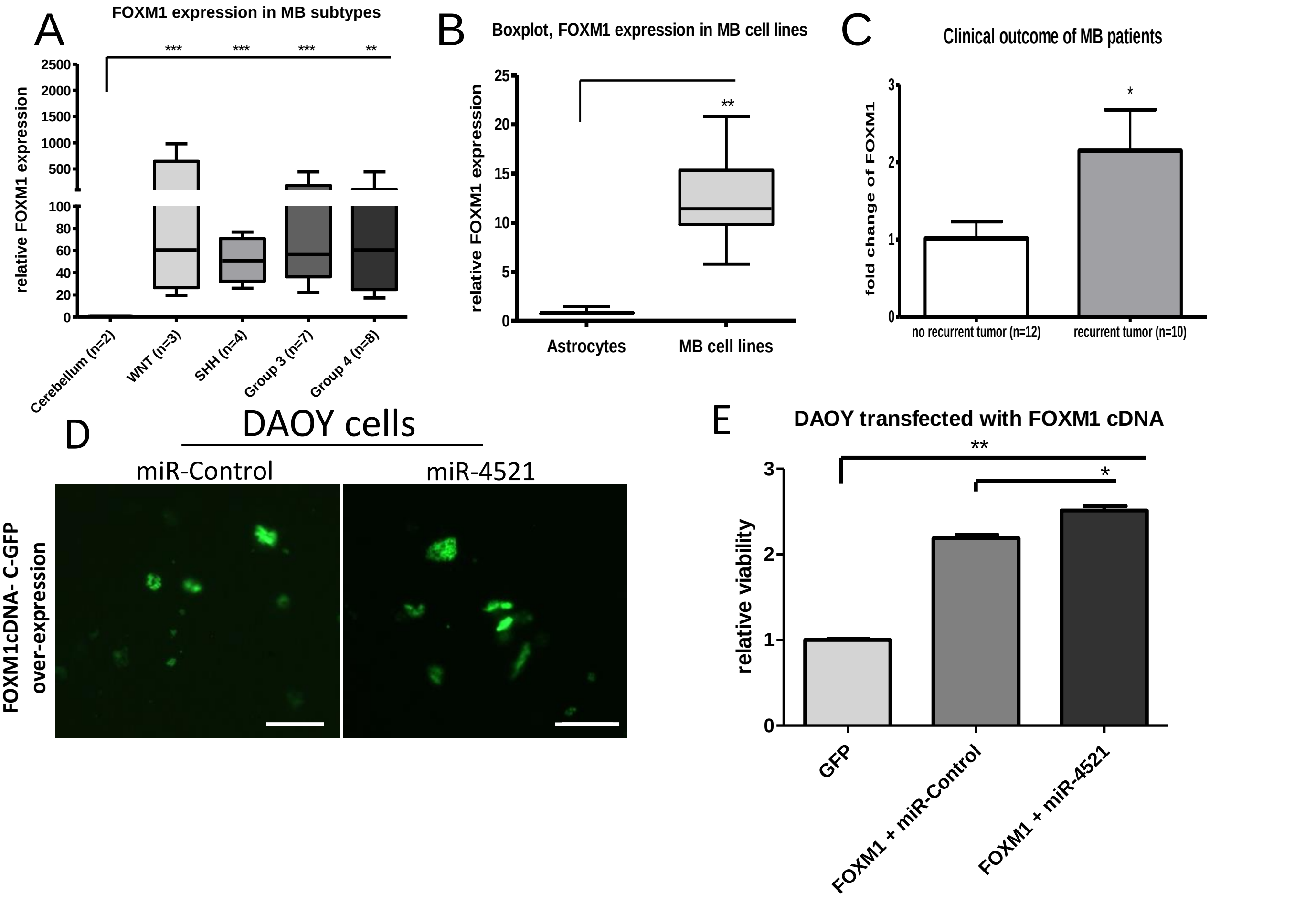

Supplementary Figure 1: mRNA expression levels of FOXM1 in MB tissue samples and MB cell lines: A) The different patients were grouped according to their molecular subtype. **B)** Box plot of FOXM1 expression in MB cell lines compared to normal astrocytes. Data were normalized to GAPDH levels. **C)** Clinical outcome of patients treated at the Medical University of Vienna. Transfection control of FOXM1-GFP cDNA overexpression combined with miR-control or miR-4521 in **D)** DAOY cells (scale bar 500µm). **E)** Growth analysis of FOXM1-GFP overexpressed cDNA plasmid in DAOY cells co-transfected with miRNAs. Proliferation was analyzed 72h after transfection. GFP plasmid was used as control. **F)** Western blot results of FOXM1 overexpression in DAOY cells. All experiments were performed in triplicates and Asterisks indicate significance (Mann-Whitney test; *P < 0.05, ***P < 0.001 or students t-test *P < 0.05, **P < 0.001), error bars indicate mean ± S.D.

Supplementary Figure 2

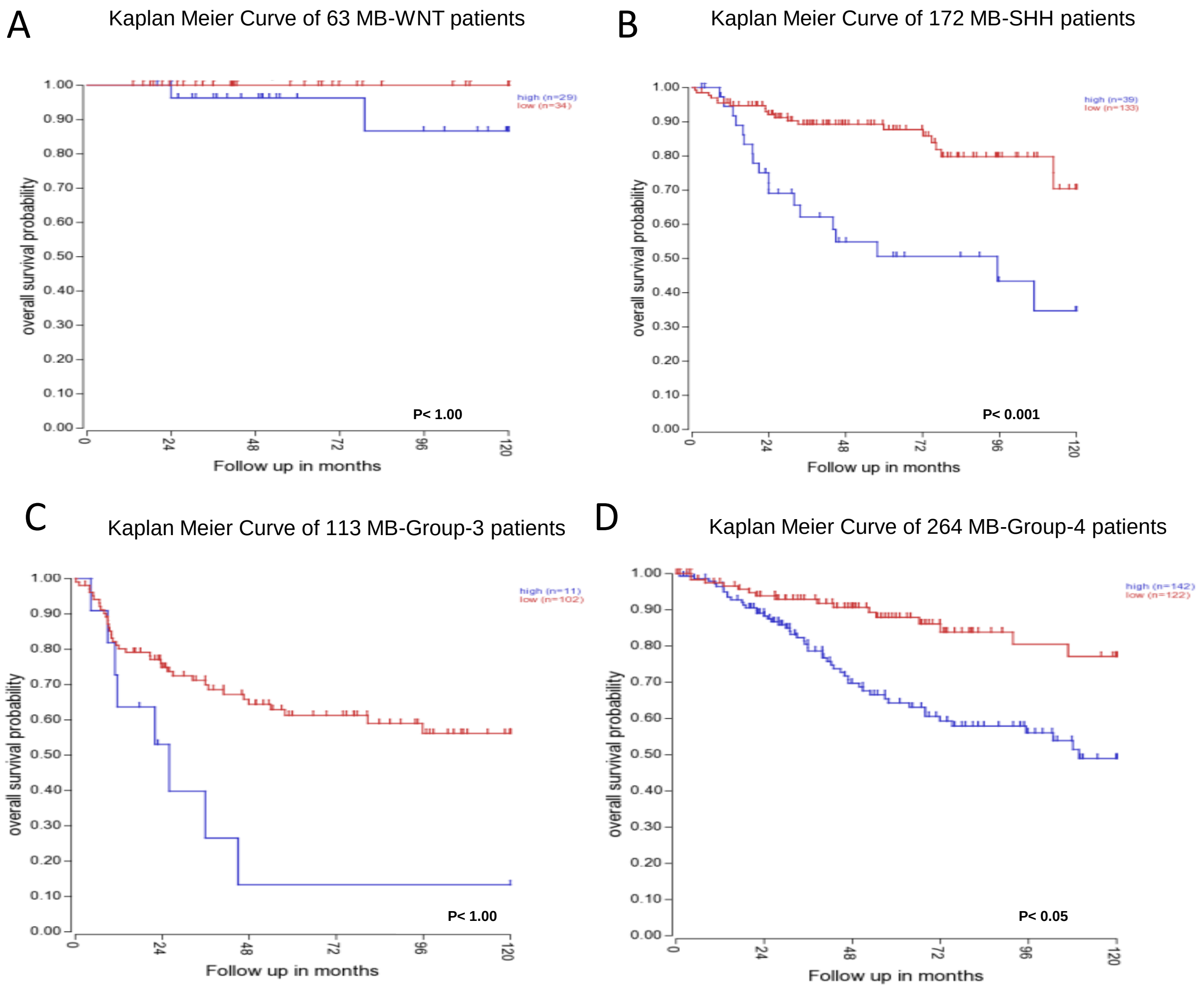

Supplementary Figure 2: Kaplan Meier curve of FOXM1 expression: 612 MB patients of Cavalli dataset hugene 11t (R2: Genomics Analysis and Visualization Platform) were split into the four molecular subgroups. **A)** WNT, **B)** SHH, **C)** group 3 and **D**) group 4. **E)** Pogression free survival Kaplan Meier curve of miR-4521 expression in our MB patient cohort (n=22).

Supplementary Figure 3

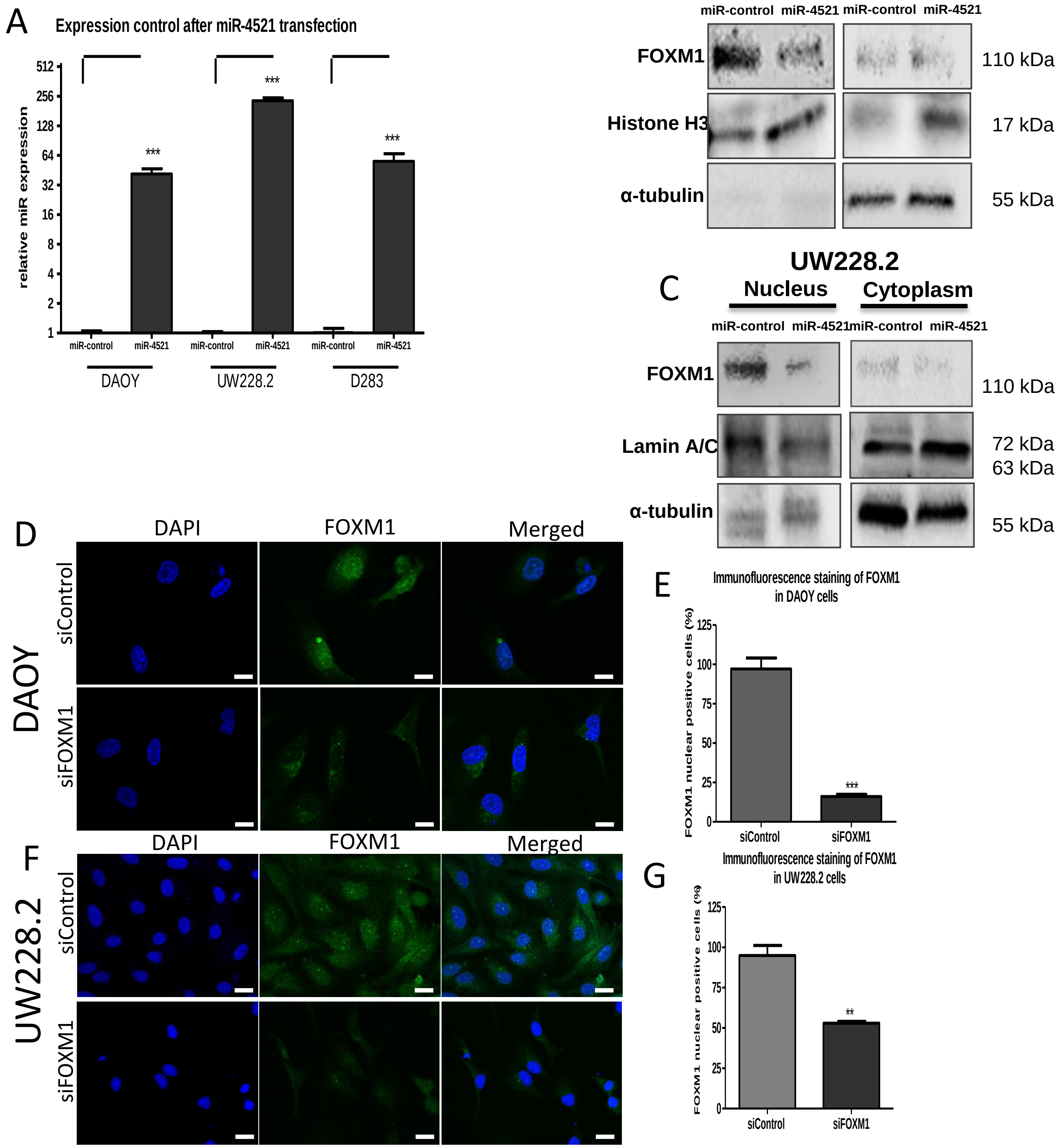

Supplementary Figure 3: Transfection control and nucleus-cytoplasm fraction of transfected MB cells and nuclear localizsation of FOXM1: A) A qPCR was performed for miR-4521 and RNU6B to determine the transfection efficacy of miR-4521 transfected MB cells. **B)** DAOY and **C)** UW228.2 cells were transfected with miR-4521 or miR-control and protein was isolated and loaded on electrophoresis gels. The levels of FOXM1 were detected via WB, Histone H3 or Lamin A/C was used as nucleus fraction control and α-tubulin for cytoplasm control. Si-Control or si-FOXM1 transfected **D)** DAOY or **F)** UW228.2 cells were fixed 72 hours after transfection and immunostained for FOXM1 (green), DNA was stained with DAPI (scale bar 20µm). **E)** and **G)** Cells showing a complete translocation from nucleus to cytoplasm. Asterisks indicate significance (students t-test; $^{**}P < 0.01$, $^{***}P < 0.001$), error bars indicate mean ± S.D.

Supplementary Figure 4

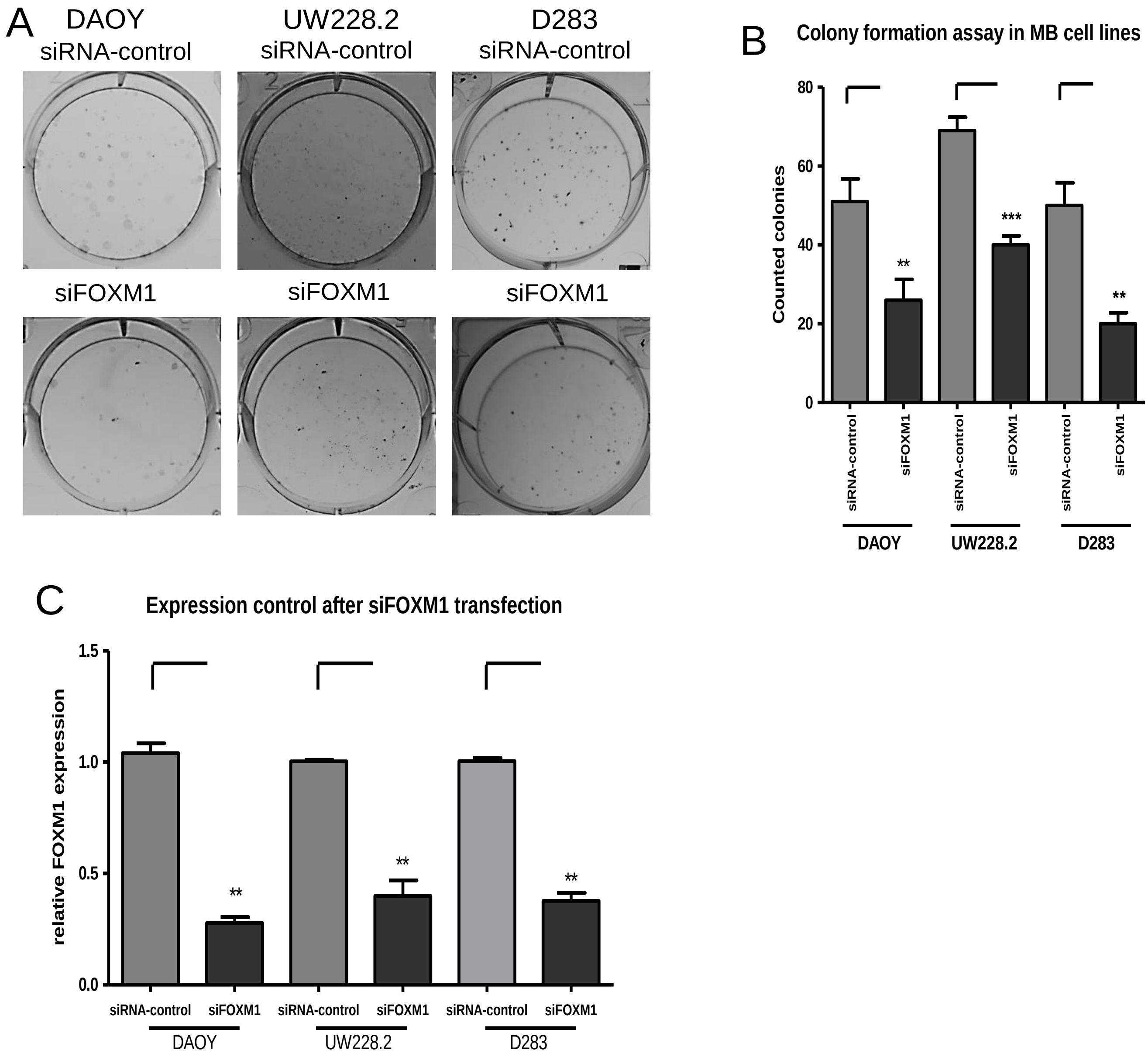

Supplementary Figure 4: miR-4521 transfection efficacy and colony formation assay using siFOXM1 transfected MB cells: A) A colony formation assay using siFOXM1 transfected MB cells was performed (1*103cells/6-well). Representative pictures of the fixed and stained cells after 7 days growing. **B)** The colonies of different MB cell lines transfected with siFOXM1 or siRNA-control were counted. **C)** A qPCR was performed for FOXM1 and GAPDH to determine the transfection efficacy of siFOXM1 transfected MB cells. All experiments were performed in triplicates. Asterisks indicate significance (students t-test; *P < 0.05, **P < 0.01, ***P < 0.001), error bars indicate mean ± S.D.

Supplementary Figure 5

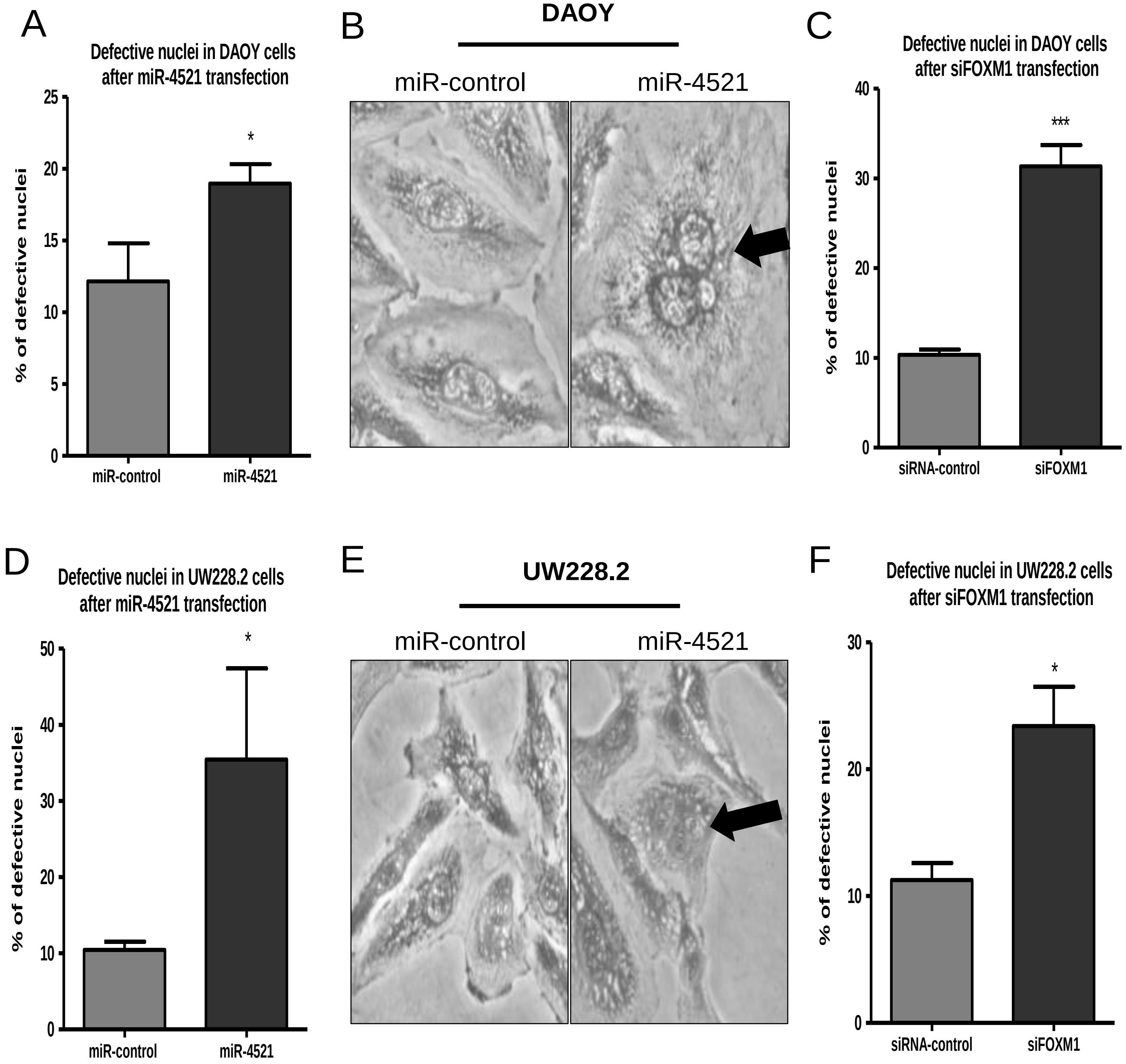

Supplementary Figure 5: miR-4521 transfected cells show elevated number of multinucleated cells: **A)** The percentage of defective nuclei was calculated in miR-4521 transfected DAOY cells at least 100 cells were counted. **B)** The percentage of defective nuclei was calculated in siFOXM1 transfected DAOY cells at least 100 cells were counted. **C)** miR-4521 transfected DAOY cells are stained with Giemsa (Arrow indicate multinucleated cells). **D)** The percentage of defective nuclei was calculated in miR-4521 transfected UW228.2 cells at least 100 cells were counted. **E)** The percentage of defective nuclei was calculated in miR-4521 transfected UW228.2 cells at least 100 cells were counted. **F)** miR-4521 transfected UW228.2 cells are stained with Giemsa (Arrow indicate multinucleated cells). All experiments were performed in triplicates. Asterisks indicate significance (students t-test; *P < 0.05, ***P < 0.001), error bars indicate mean ± S.D.

Supplementary Figure 6

miR-control miR-4521

DAOY

UW228.2

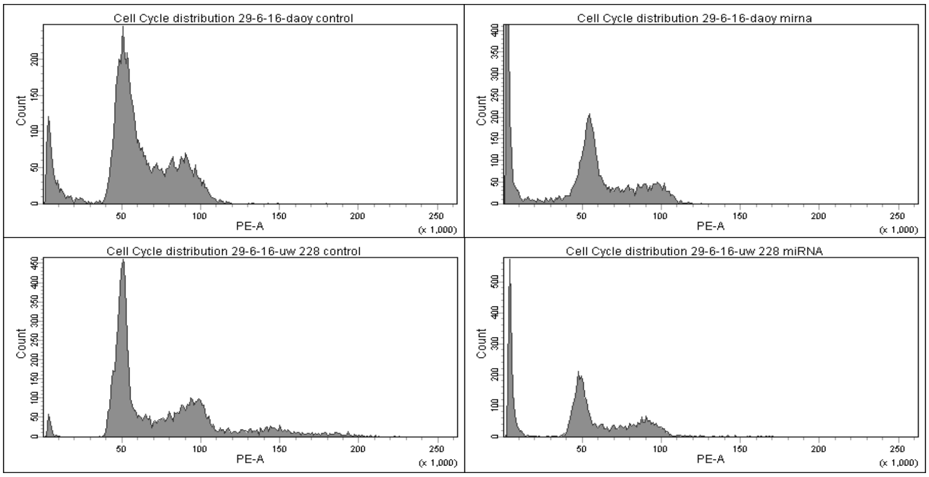

%	miRNA control subG1	G1	S	G2/M	miRNA 4521 subG1	G1	S	G2/M
**DAOY**	7,5	29,6	9,3	11,4	20,9	22,7	6,6	9,7
**UW228.2**	2	41	10,1	17,8	16,9	21,1	6,9	8,6

Supplementary Figure 6: Cell cycle distribution of the miR-4521 transfected MB cells. miR-control or miR-4521 transfected MB cells were stained with PI solution and cell cycle distribution was measured by using a FACS Canto II and analyzed with the FACS Diva software.

Supplementary Figure 7

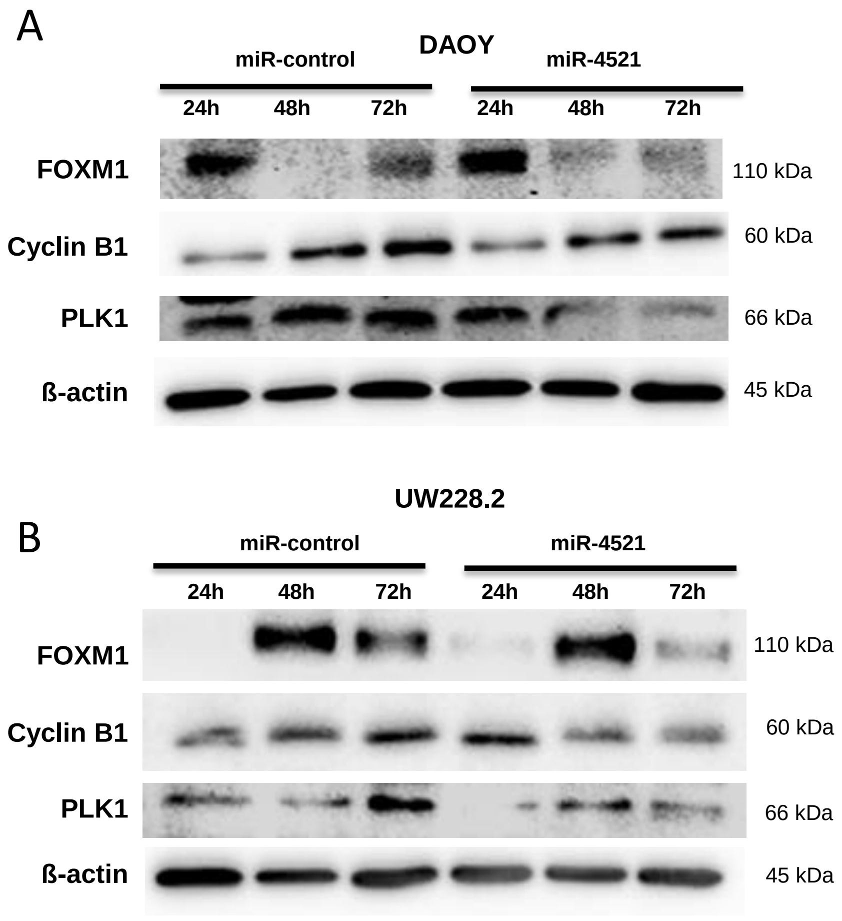

Supplementary Figure 7: Time curve of FOXM1 downstream targets. Protein of miR-control or miR-4521 transfected **A)** DAOY and **B)** UW228.2 cells was harvested after 24, 48 and 72 hours. Wester Blot analysis was performed. Each sample contains 10µg protein and β-actin was used as loading control.

Supplementary Figure 8

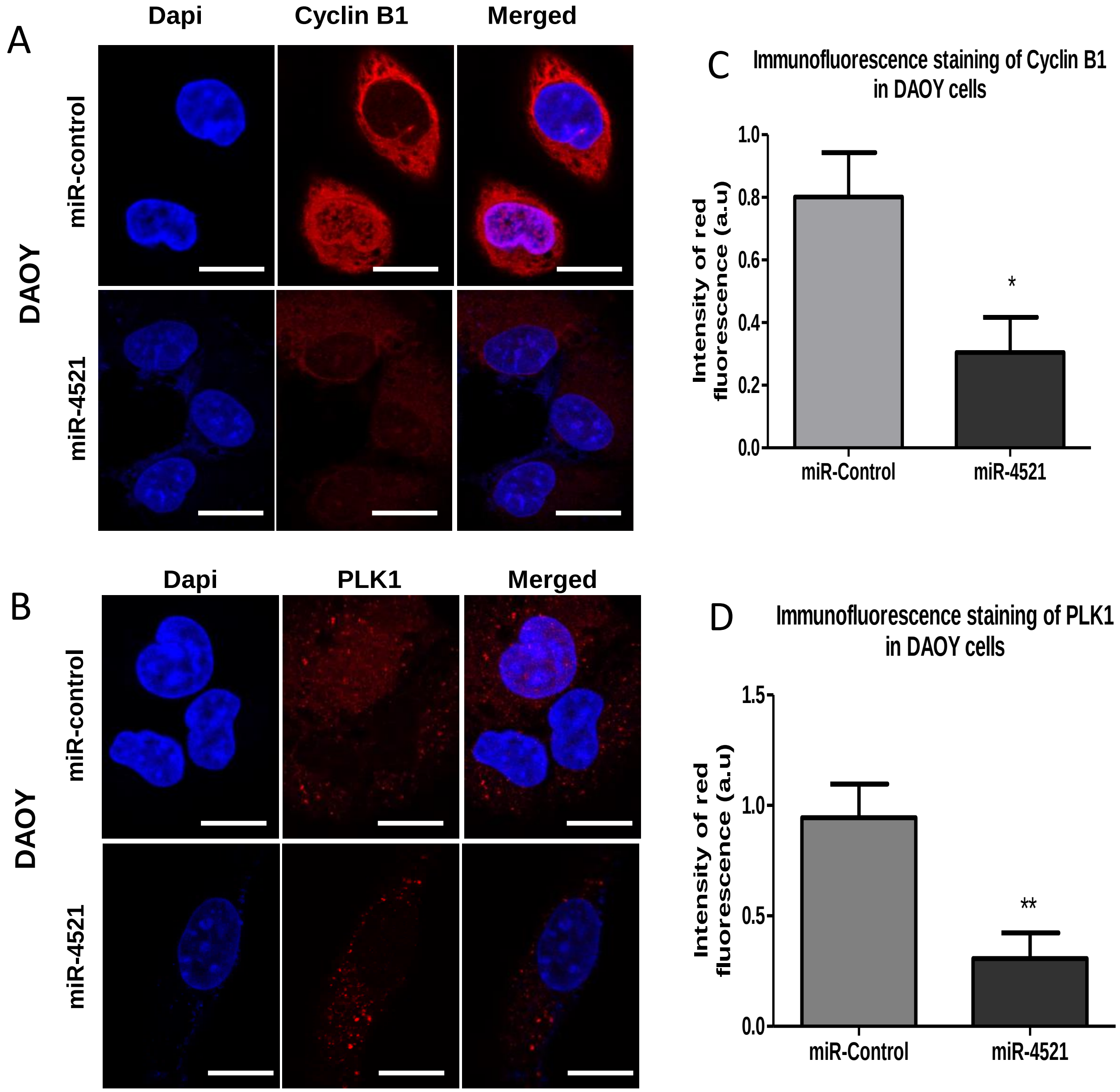

E

	FOXM1	*CCNB1*
FOXM1	1	
CCNB1	0,594375	1

F

	FOXM1	*PLK1*
FOXM1	1	
PLK1	0,670015	1

Supplementary Figure 8: Confocal microscopy of downstream targets of FOXM1. miR-control or miR-4521 transfected DAOY cells were fixed 72 hours after transfection and immunostained for **A)** Cyclin B1 (red) or **B)** PLK1 (red), DNA was stained with DAPI (scale bar 20µm). Intensity of **C)** Cyclin B1 or **D)** PLK1 fluorescence was calculated according to following formula: corrected total cell fluorescence = Integrated Density – (area of selected cell x mean fluorescence of background readings). The analysis was performed using Image J Software Correlation of FOXM1 gene and **E)** CCNB1 or **F)** PLK1 in Cavalli dataset hugene 11t (R2: Genomics Analysis and Visualization Platform). All experiments were performed in triplicates. Asterisks indicate significance (students t-test; *P < 0.05, **P < 0.01), error bars indicate mean ± S.D.

Supplementary Figure 9

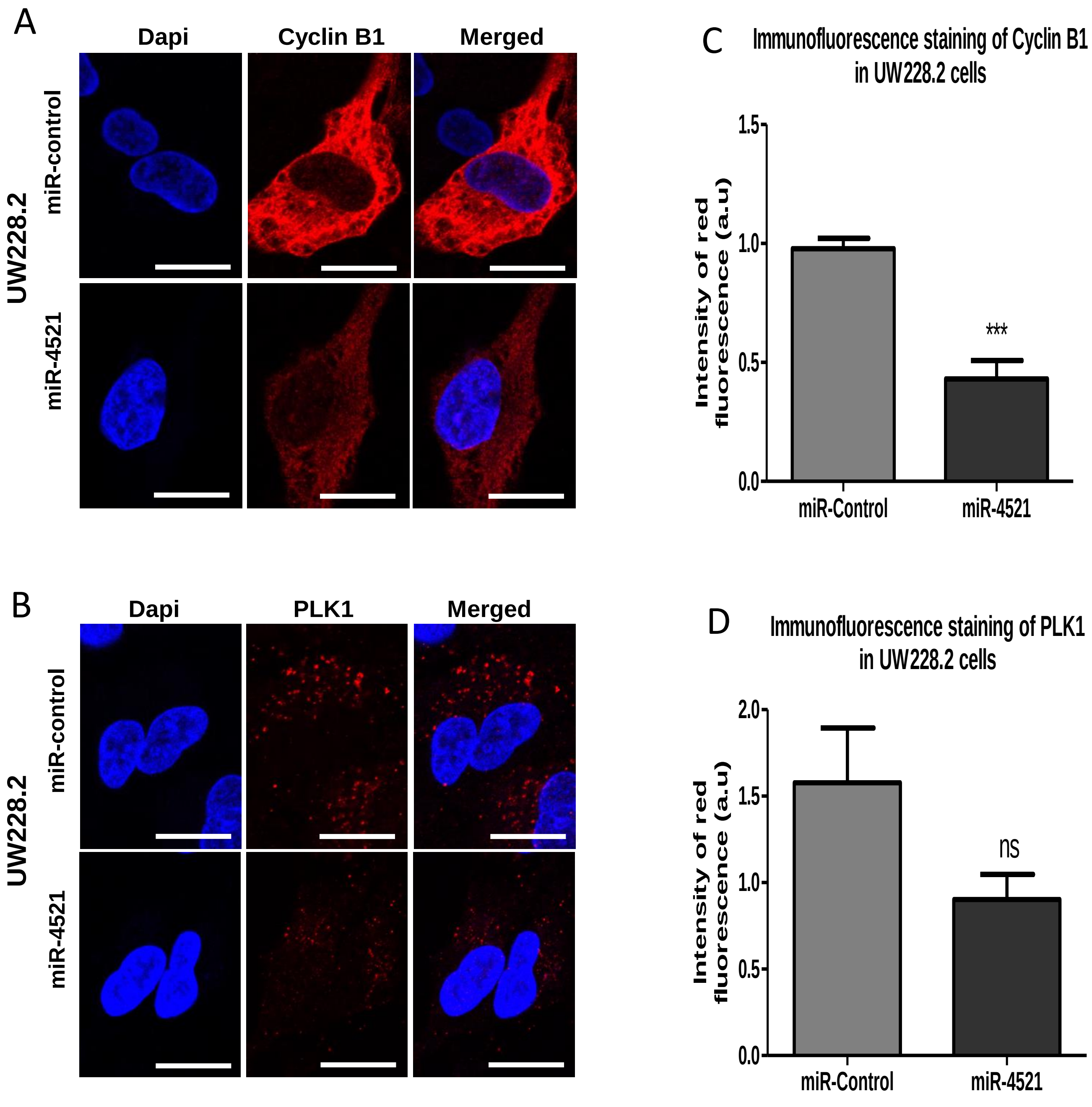

Supplementary Figure 9: Confocal microscopy of downstream targets of FOXM1. miR-control or miR-4521 transfected UW228.2 cells were fixed 72 hours after transfection and immunostained for **A)** Cyclin B1 (red) or **B)** PLK1 (red), DNA was stained with DAPI (scale bar 20µm). Intensity of **C)** Cyclin B1 or **D)** PLK1 fluorescence was calculated according to following formula: corrected total cell fluorescence = Integrated Density – (area of selected cell x mean fluorescence of background readings). The analysis was performed using Image J Software. Asterisks indicate significance (students t-test; ns = no significance, ***P < 0.001), error bars indicate mean ± S.D.
