# Supplementary material for: High impact of miRNA-4521 on FOXM1 expression in medulloblastoma
Source: Cell Death Dis. 2019 Sep 20;10(10):696. doi: 10.1038/s41419-019-1926-1 (PMC6754377; doi:10.1038/s41419-019-1926-1)
Supplement: Supplementary file 2 — Supplementary file [file 41419_2019_1926_MOESM2_ESM.docx]

**Supplementary experiemental procedures:**

**Real-time PCR**

RNA was isolated using Tri reagent (Sigma-Aldrich) and for detection of the miRNA the TaqMan system was chosen.10 ng RNA was used for reverse transcription using the TaqMan MicroRNA RT Kit (Applied Biosystems, Life Technologies). For the detection of FOXM1 and GAPDH the SYBR-green system was used. Quantitative real-time PCR of miR-4521 and RNU6B were performed in triplicates using a StepOnePlus System (Life Technologies). The miRNA levels were normalized to the stable internal control miRNA RNU6B. For the detection of FOXM1 and GAPDH the SYBR-green system was chosen and equal amount of total RNA samples (1 µg for cells/tumor tissue) were reverse-transcribed by using the High-Capacity cDNA Synthesis Kit (Applied Biosystems, #4368814). All quantitative real-time PCRs were performed using the GoTaq qPCR Master Mix (Promega, A6001) on a StepOnePlus Real-Time PCR System (Applied Biosystems). Relative fold changes between expression of target genes in tumor and control samples were calculated by using the 2-ddCq method. GAPDH was used as a housekeeping gene to normalize gene expression. As a control a commercial available medley of healthy cerebellum (Clontech, #636530, pooled from three Asian males aged 21-29) was used. The following primer sequences ordered from IDT (Integrated DNA Technology) were used in this manuscript:

FOXM1: forward 5`- TGCCCAGCAGTCTCTTACCT-3`

reverse 5`-CTACCCACCTTCTGGCAGTC-3`,

GAPDH: forward5`- ACATCGCTCAGACACCATG-3`

reverse 5`- TGTAGTTGAGGTCAATGAAGGG-3`

**Nucleus-cytoplasm fraction**

The miR-4521 and control transfected cells were harvested 3 days post transfection, washed twice in PBS and and lysed in buffer A (10mM HEPES, 10 mM KCl, 0.1 mM EDTA, 0.1 mM EGTA, 1 mM DTT, 1.5% NP-40 and 1.5% PMSF) on ice for 10 minutes followed by centrifugation at 12000 rpm for 10 minutes. Supernatants were collected as cytoplasmic extracts.

The remaining pellets were washed in PBS and re-suspended in buffer B (20 mM HEPES, 400 mM NaCl, 1 mM EDTA, 1 mM EGTA, 1 mM DTT, 0.05% NP-40, 0.05% PIM, 0.05% PMSF) and homogenized at 4°C for 20 minutes. After centrifugation at 12000 rpm, the supernatants were collected as nuclear extracts. The extracts were analyzed by Western blotting.

**Spheroid invasion assay**

For creating spheroids the hanging drop method was performed. Therefore, the miR-4521 transfected cells were suspended in media containing 0.3% methylcellulose. Drops of 30 µl of the suspension (5000 cells) were distributed equally over a 10 cm dish. The plates were incubated upside down for two days in the incubator to allow the formation of stable spheroids. Afterwards all hanging drop were collected into a 50ml falcon tube and embedded into 1.5% rat tail collagen gels (Corning, Cat no:354236). To prepare the collagen solution according to the manufacturer’s protocol, 3% collagen solution were prepared and mixed with an equal volume of 0.85% (w/v) methylcellulose containing Dulbecco’s Modified Eagle Medium from Sigma-Aldrich, and 10% FBS (Sigma-Aldrich ). The spheroid suspension was pipetted into 24-well plates (350µl/well) and placed into the incubator for 30 minutes for polymerization. For stimulation, collagen gels were overlaid with medium containing 0.5% FBS and then incubated at 37°C, 5% CO_2_ with 100% humidity. Quantification of sprouting intensity was determined by the cumulative sprout length per spheroid using Nikon inverted phase-contrast microscope and Image J software converting pixels to micrometers. For each experiment at least 10 spheroids were quantified.

**Immunohistochemistry**

Formalin-fixed, paraffin-embedded tissue sections (3µm) were used for immunohistochemistry. FOXM1 immunohistochemical staining was performed manually. Briefly, sections were pre-treated with Tris/EDTA pH9 buffer (K8004 target retrieval solution high pH; Agilent Technologies/Dako, Santa Clara, United States) for 20 minutes at 95°C. Then, endogenous peroxidase was blocked with Dako REAL™ Peroxidase-Blocking Solution (S2023; Agilent Technologies/Dako, Santa Clara) for 10 minutes at room temperature. Polyclonal rabbit FOXM1 antibody was diluted at 1:100 and sections were incubated overnight at 4°C. For detection and visualization Dako REAL™ EnVision™ Detection System (K5007, Agilent Technologies/Dako) was used according to manufacturer’s instructions with diaminobenzidine tetrahydrochloride (DAB) as chromogen. Finally, sections were counterstained with Mayer's hemalum, differentiated, dehydrated, cleared in n-Butylacetate and mounted. In total, 1000 cells of each slide were manually counted and the percentage of FOXM1 positive stained nuclei was calculated.
